# Chinese Children with Congenital and Acquired Blindness Represent Concrete Concepts in Vertical Space through Tactile Perception

**DOI:** 10.3390/ijerph191711055

**Published:** 2022-09-03

**Authors:** Guangyin Shen, Ruiming Wang, Mengru Yang, Jiushu Xie

**Affiliations:** 1 Shenzhen Yuanping Special Education School, Shenzhen 518112, China; 2 Key Laboratory of Brain, Cognition and Education Sciences, Ministry of Education, School of Psychology, Center for Studies of Psychological Application, and Guangdong Key Laboratory of Mental Health and Cognitive Science, South China Normal University, Guangzhou 510631, China; 3 School of Psychology, Nanjing Normal University, Nanjing 210097, China

**Keywords:** Chinese children, concrete concept, spatial representation, grounded cognition, tactile sensory processing, behavioral experiment

## Abstract

Many studies have tested perceptual symbols in conceptual processing and found that perceptual symbols contain experiences from multisensory channels. However, whether the disability of one sensory channel affects the processing of the perceptual symbols and then affects conceptual processing is still unknown. This line of research would extend the perceptual symbol theory and have implications for language rehabilitation and mental health for people with disabilities. Therefore, the present study filled in this gap and tested whether Chinese children with congenital and acquired blindness have difficulty in recruiting perceptual symbols in the processing of concrete concepts. Experiment 1 used the word-pair-matching paradigm to test whether blind children used vertical space information in understanding concrete word pairs. Experiment 2 used the word-card-pairing paradigm to test the role of tactile experiences in the processing of concrete concepts for blind children. Results found that blind children automatically activated the spatial information of referents in the processing of concepts through the tactile sensory channel even when the visual sensory channel was disabled. This finding supported the compensatory phenomenon of other sensory channels in conceptual representation. In addition, the difference between elementary school blind children and middle school blind children in judging the spatial position of concrete words also indicated the vital influence of perceptual experiences on perceptual symbols in conceptual representation. Interestingly, there were no significant differences between children with congenital or acquired blindness. This might suggest that the compensatory of other sensory channels did not have a sensitive period. This study not only provided new evidence for the perceptual symbol theory but also found that perceptual symbols could be developed by a compensatory mechanism. This compensatory mechanism can be used to develop a rehabilitation program for improving language learning in blind children. Improved language ability in blind children will also improve their mental health problems caused by difficulties in social interaction (e.g., social anxiety).

## 1. Introduction

Language learning is a remarkable ability for humankind. Concepts are the basis of the human cognitive system. In particular, concrete concepts are frequently used in our daily life. Concrete concepts usually refer to concrete things in daily life. Therefore, learning concrete concepts is important for language learning. Difficulty in understanding and using concrete concepts in communication may result in social difficulties and even mental health problems (e.g., social anxiety disorder). Hence, this line of research has significant theoretical and practical implications, especially for people with language-learning difficulties (e.g., blind people).

One vital aim of language learning is to build mental representation of objects, events or ideas that are described by words. Representation is the internal representation of external things in psychological activities. Concept representation refers to the acquisition and extraction of conceptual knowledge, which determines the way people understand concepts [1]. Therefore, how people conceptually represent the object is the core issue that has been debated in the field of cognitive research.

### 1.1. Theories of Conceptual Representation

To date, there is still a heated debate surrounding the form of conceptual representation. The traditional propositional symbol theory emphasizes that concepts are represented in a propositional manner and stored in linguistic memory as abstract signs, independent of the subject’s perceptual, motor, and affective systems. The propositions also form a propositional network for processing information due to their interconnectedness [2,3]. This theory also has strong explanatory power precisely because of the infinite capacity of the propositional network, but it has also been controversial because of the lack of direct empirical evidence to support it.

Given the deficiencies of propositional symbol theory, Barsalou proposed a new conceptual representation theory—perceptual symbol theory. The theory holds that the concept representation system and the perceptual motor system have overlapped neural bases. Conceptual processing and perceptual processing frequently interact to support language processing and learning. Concepts are essentially neural recordings of perceptual and motorial experiences in subject experience objects. The basic form of concept processing is the simulation of bodily experience [4]. The emergence of perceptual symbol theory did not completely negate propositional symbol theory. However, perceptual symbol theory provided a completely new approach for understanding conceptual representation and indirectly promoted the emergence of following related theories. Furthermore, it also improved the practical application of language learning, information processing, and other fields. For example, motor action has been used to improve people’s performance in retrieving emotional memories [5].

### 1.2. Perceptual Symbol Theory

Perceptual symbol theory has received a lot of research support. This theory holds that the basis of concept processing is sensorimotor; that is, phenomena that exist in sensory processing also appear in concept processing [1]. To test this hypothesis, Pecher et al. [6] designed an experimental study of conceptual feature recognition based on the switching cost. During the process, concepts and attribute pairs were presented on the screen in turn, and participants were asked to judge whether a feature was a common attribute of a concept. The results showed that when the first presented concept and the latter concept did not belong to the same sensory channel, the response time of the participants would increase, and the correct rate would decrease, showing a switching cost effect. This experiment is also the first to demonstrate that concept processing is based on sensorimotor representations. On this basis, Vermeulen et al. used the same experimental paradigm to examine the switching cost of emotional concepts and found that switching cost also existed in emotional concept processing [7]. Furthermore, Van Dantzig, Pecher et al. inferred that perceptual processing would also affect concept processing based on the principle of conversion consumption and proposed a perceptual interference experimental paradigm. In this experiment, experimental instruments were used to provide the participants with auditory, visual, and tactile information. The participants first performed a perceptual task, which required them to judge the left and right directions of the stimulus as soon as possible. When performing the concept attribute judgment task, the participants needed to judge whether the attributes presented on the screen belonged to the concepts of the presented words. The experiment found that when the sensory and conceptual channels involved in the perceptual stimulus were the same, the participants judged the concept attribute faster. Their experiments demonstrated that conceptual processing and perceptual processing have the same properties [8]. Based on the above research, Vermeulen et al. proposed the sensory load paradigm. This paradigm assumed that when a perceptual channel was occupied, it became difficult for the brain to process concepts that contain perceptual information from this channel. In the experiment, the participants first memorized two types of perceptual stimuli, such as one or three meaningless sounds and meaningless gray graphics, and then judged whether a concept presented on the screen matched an attribute after the memory was completed. Immediately afterward, one or three perceptual stimuli that had been presented or not presented before would appear on the screen, and the participants needed to judge whether these stimuli had appeared in the previous judgment task based on memory [9]. The above results finally showed that concept processing is a process involving both sensation and perception. Under the condition of a certain channel load, the difficulty of processing a concept containing this channel information will increase.

### 1.3. Spatial Consistency Effect

Given the importance of specific concepts in language acquisition and concept representation, previous studies focused on the perceptual symbolic representation of concrete concepts. Among them, many researchers used the spatial consistency effect to experimentally test the perceptual symbol representation of concrete concepts. Simon and Small proposed the Simon effect; that is, when the physical location of the stimulus corresponds to the location of the desired response, people respond faster and more accurately to the non-spatial properties of the stimulus [10]. Based on the above logic, Zwaan and Yaxley proposed the famous word-pair-matching paradigm experiment. The experiment was integrated with the visual field separation technique in neuroscience, and participants were asked to judge word pairs that implied vertical spatial positional relationships. Taking the words “roof” and “floor” for example, if the word roof is presented above the word floor, the participants will recognize it faster and more accurately. If the position is opposite, the response time of the participants will be longer, and the accuracy rate will also decrease. The success of the word-pair-matching paradigm experiment more closely supported the idea that perceptual symbols arise in the right hemisphere [11]. This finding was also replicated and extended in Chinese. Wang first replicated and improved Zwaan’s experiment and showed that the spatial consistency effect would also appear in Chinese speakers. On this basis, the perceptual symbol theory was further tested by examining whether the spatial distance effect occurred in the semantic correlation judgment task [12]. At present, in the deeper research on the symbolic representation of concrete concept perception, researchers also found that the spatial consistency effect can be used in the horizontal direction to prove the correspondence between concrete concepts and abstract concepts. Specifically, Ouellet et al. proposed a cue paradigm experiment, which used people’s perception of temporal representation from left to right. At the beginning of the experiment, a word was presented in the center of the screen, and the participants responded to the direction presented by the origin on the screen later. After judging the position of the box, an answer was given concerning the temporal meaning contained in the words presented at the beginning. The results showed that participants responded faster to the dots that appeared on the right when the word in front of them expressed the meaning of the future. At the same time, the previously presented past words would speed up the response to the left dot. This experiment proved that humans would associate abstract concepts with concrete concepts for cognition and need to use perceptual knowledge to understand abstract concepts [13].

In conclusion, previous studies validated perceptual symbol representations in concrete concept representations. Moreover, the theory of perceptual symbols holds that perceptual symbols originate from perceptual experience. Perceptual experience indicates that sensory and perceptual states are acquired from different sensory and perceptual channels, such as visual channel and tactile channel. Hence, for individuals with deficits in perceptual pathways, how do they construct perceptual symbol representations? Specifically, can congenitally blind patients still establish perceptual symbol representations in the absence of visual sensory channel? After acquired blindness loses the visual sensory channel, do they still use the existing or establish a new perceptual symbol representation? If there are perceptual symbol representations in blind patients, is there a difference in perceptual experience between congenitally blind patients and acquired blindness? Are there differences between congenitally blind patients and acquired blindness patients at different ages? The theory of perceptual symbols does not provide a clear theoretical perspective on the above questions.

### 1.4. Research on Conceptual Representation of Blind People

The theory of perceptual symbols holds that the perceptual-motor system plays an irreplaceable role in the acquisition of concepts [14]. Conceptual knowledge is formed based on the subject’s perceptual-motor experience. Perceptual motor experience is the beginning of the subject’s understanding of the world and constitutes the content of the conceptual representation. In the process of individual understanding and processing of concepts, the restoration of perceptual-motor experience is the basic way [15]. Since the establishment of perceptual symbols originates from people’s direct perceptual experience, when people lose the perceptual information of a certain channel, people also lose their direct perceptual experience. This can lead to people being unable to continue building perceptual symbol representations.

However, there is another opposite possibility. According to the irreplaceable inference of the perceptual motor system for concept acquisition, as long as the perceptual system exists, individuals can still construct perceptual symbol representations. Perceptual symbol representation is a comprehensive representation system that contains multi-channel information from different perceptual channels [16]. This indicates that people with disabilities can compensate for the lack of sensory information input through the non-impaired perceptual channels. Specifically, even if blind patients lose their visual channel, external stimuli can still be processed by cognitive systems through perceptual channels such as hearing, smell, and touch to complete the conceptual representation.

In recent years, many researchers have begun to pay attention to the blind group and conduct a shallow exploration of their conceptual representation. Saysani found in a study that people with congenital blindness can still have color knowledge similar to ordinary people and have a similar color wheel concept even if they do not have visual sensory channel [17]. Bedny et al. found that blind people and ordinary people are similar concerning understanding words through word-sense similarity scores, although they lack the information transmission of visual sensory channel. These studies all indirectly suggest the existence of symbolic representations of perception in blind people but do not explore them further [18]. In addition, the study of Crollen et al. showed the importance of tactile sensory channel for information reception and integration of blind people [19]. Blind people can integrate internal and external information through tactile positioning, and even congenitally blind people can adjust spatial integration through top-down information. For blind people, internal body coordinates are an important reference for thinking about spatial relationships, while the tactile sensory channel is important for transforming and integrating external spatial information and establishing external coordinates. Based on the above viewpoints, this study believes that blind people can still express perceptually through the tactile sensory channel in the absence of visual channels, and the tactile sensory channel plays an equally important role as visual channels in the representation of concepts.

### 1.5. Research Questions

To find out whether blind people can construct perceptual symbol representations and the influence of perceptual experience on blind people’s language processing, this study carried out two experiments using concrete concept words as research materials. Findings of present study will shed light on the above-mentioned debates between propositional symbol theory and perceptual symbol theory. The present study focuses on two research questions:

**RQ1.** Whether children with congenital and acquired blindness establish perceptual symbols of concrete words after losing the visual sensory inputs.
**RQ2.** Whether tactile experience plays a compensatory role in conceptual representation for blind children.

In Experiment 1, a paper-and-pencil test was used to allow blind children to judge whether the implied spatial relationship between two concrete words above and below on a card was consistent and choose the answer sheet. If the judgment results showed that the participants were indeed affected by the implied spatial position of word pairs, they may suggest that blind children still automatically activate the spatial information of referents through the tactile sensory channel for knowledge representation when their visual sensory channel is blocked. This further showed the compensation phenomenon of other sensory channels (e.g., tactile sensory channel) among blind children. Furthermore, Experiment 2 tested whether the perceptual experience had a key influence on the knowledge representation of totally blind students. In Experiment 2, blind students moved the Braille cards with concrete words to corresponding positions by judging the positional relationship between alternative and reference words. Experiment 2 used a more complex task than Experiment 1. In Experiment 2, participants needed to make further choices from alternative word cards and put them into the correct position. Therefore, if the results showed that the implied spatial position of word pairs also modulated participants’ responses, they suggested that the experience obtained and accumulated through the tactile channel plays an important role in the knowledge representation of blind students. Perceptual experience, especially the tactile experience, has a certain impact on the knowledge representation of completely blind students. Exploring the above questions is not only of great theoretical significance for revealing the mechanism of perceptual symbol theory, but also has important practical significance for understanding the language-learning mechanism of visually impaired people and providing more reference theoretical bases for language rehabilitation activities.

## 2. Experiment 1

### 2.1. Purpose

The purpose was to explore whether blind students with congenital blindness and acquired blindness automatically activate the spatial information implied by words through the tactile sensory channel.

### 2.2. Methods

#### 2.2.1. Participants

In this experiment, according to the selection criteria of the participants—normal intelligence, ability to read and write Braille, and absolute blindness—a total of 132 blind students from Shenzhen Yuanping Special Education School, Nanjing School for the Blind, Guangzhou School for the Blind, Beijing School for the Blind, and Tianjin School for the Visually Impaired were recruited to participate in this experiment. Specifically, there were 80 congenitally blind students (44 boys, the mean age was 12.8), including 34 blind students (19 boys, the mean age was 9.4) in the lower grades of primary schools and 46 blind students (25 boys, the mean age was 16.2) in junior high schools and above. There were 52 acquired blind students (30 boys, the mean age was 12.65), including 24 (14 boys, the mean age was 9.6) in the lower grades of primary schools and 28 blind students (16 boys, the mean age was 15.7) in junior high schools and above. Acquired blindness was mostly caused by medical malpractice, chronic eye disease, and accidental injury. Their mean age of blindness was 3. All the test participants are native speakers of Mandarin Chinese, can read and write in Braille, have no other disabilities except blindness, and can understand the experimental procedures normally. Before the experiment was carried out, the consent of the blind students themselves, teachers, or guardians was obtained, and the experimental data were kept strictly confidential.

#### 2.2.2. Experimental Design

A mixed experimental design of 2 (position relationship: consistent vs. inconsistent) × 2 (grade: lower elementary school vs. junior high school and above) × 2 (participant type: congenital blind vs. acquired blindness) was adopted, where the intra-group variable was spatial relationships, the between-group variables were grade and participant type, and the dependent variable was the correct rate of participants’ responses.

#### 2.2.3. Experimental Materials

The experimental materials were 64 pairs of two-character concrete nouns. All experiment words were selected from previous studies [12,20,21,22,23,24,25,26,27].

**Figure 1:**
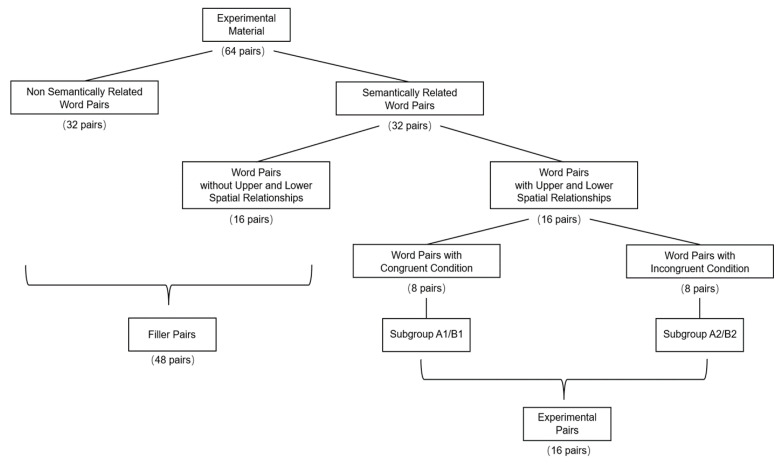
The allocation of materials in Experiment 1.

To counterbalance potential influence from the differences in materials, two material groups were created for the experiment. Each material group consisted of 64 pairs of two-character concrete nouns, which contained 16 experimental word pairs, 16 semantically related filler word pairs, and 32 semantically irrelevant filler word pairs. The 16 experimental word pairs were word pairs implying upper or lower spatial relationships and were semantically related (e.g., airplane–runway, ceiling–floor). Both material groups had the same 16 experimental word pairs. We also divided the 16-word pairs into two sub-groups, namely subgroup A and subgroup B. Each subgroup contained eight word pairs. In one group, word pairs in sub-group A were presented on the card according to the implied spatial location to create a consistent condition (e.g., airplane was presented above while runway was presented below). Word pairs in sub-group B were presented opposite their implied spatial location to create an inconsistent condition (e.g., airplane was presented below while runway was presented above). This version of experimental material was A1B2. In another counterbalanced version, word pairs in sub-group A were presented in an inconsistent way and word pairs in sub-group B were presented in a consistent way. This version of experimental material was A2B1. Two versions of experimental materials used the same word pairs and only their presentation location was manipulated. Therefore, the difficulty of the two versions was identical. This manipulation also corresponds to the independent factor of the experiment, i.e., position relationship: consistent vs. inconsistent. Participants were randomly allocated one version (i.e., A1B2 or A2B1) of experiment material. In each trial, participants judged whether two words from the word pair were semantically related. This manipulation could minimize potential influence of different experimental materials on the participants’ responses. Please refer to Figure 1 for the allocation of materials.

None of the words in the 48 filler word pairs implied spatial information. Among them, the words in the 16 semantically related word pairs can be classified into the same category and have strong semantic connections (e.g., apple–snow pear). The words in the 32 semantically irrelevant word pairs cannot be classified into one category and were not semantically correlated (e.g., flagpole–broad bean). The filler word pairs for both versions were the same. Filler words there cannot be used as “control” or baseline. If we compared critical and filler words, we actually conducted a between-item comparison. This comparison would induce potential confounding variables and could influence our findings. Adding 48 filler word pairs could ensure that there were 32 pairs of semantically related word pairs and 32 semantically unrelated word pairs in the experiment, achieving a balance of Yes and No responses. At the same time, the addition of semantically related filler word pairs can make the participants ignore the implied contextual spatial relationship information in the experimental word pairs to avoid the participants’ guessing the real purpose of the experiment.

**Figure 2:**
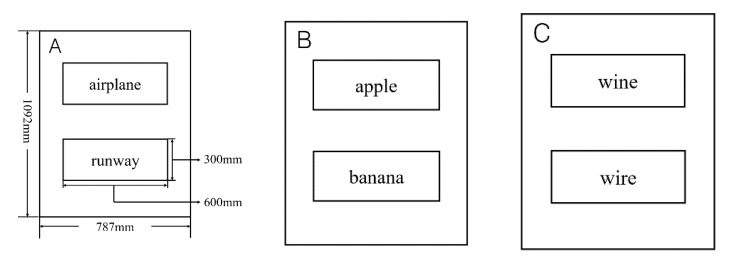

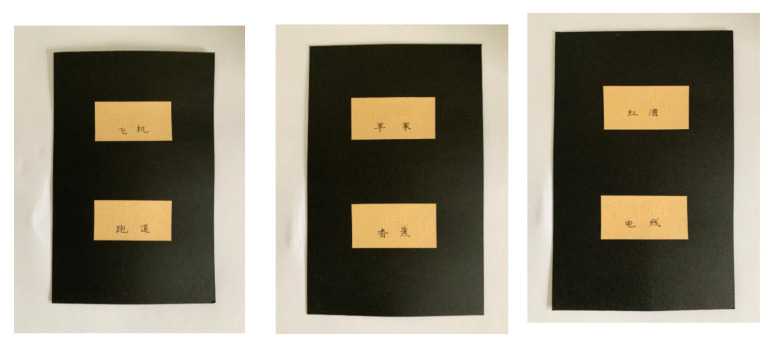
Word cards used in Experiment 1. (**A**) The agreement condition in the experimental word pair; (**B**) the semantically related filler word pair; (**C**) the semantically irrelevant filler word pair. Below the illustrations are the materials used in the experiment. Below the illustrations are the materials used in the experiment. In the three images: “飞机” means “airplane” in Chinese, “跑道” means “runway”, “苹果” means “apple”, “香蕉” means “banana”, “红酒” means “wine”, “电线” means “wire”.

Due to the visual impairment of the participants, this experiment was carried out using a pen and paper experiment. All stimuli were presented in Braille on word cards. First, we used the Braille editor BWORD software opened by China Braille Publishing House to convert all experimental application word pairs into current Braille with all scales, then used a Braille marking machine to engrave them, and cut each word into 600 × 300 mm word pieces. Finally, the two words were glued up and down symmetrically on a 787 × 1092 mm piece of cardboard to make a word card presented to the participants in the experiment. Please refer to Figure 2 for an example of a word card.

#### 2.2.4. Experimental Procedure

This research used a pen-and-paper experiment. In the experiment, all word cards would be randomly presented to the participants. The participants needed to read the words from top to bottom and judge whether the two were semantically related as soon as possible. Then they needed to mark with a pen “relevant/irrelevant” braille. Before the critical experiment, the participants would undergo a practice experiment. The experimenter simultaneously presented three cards that were used to present words to the participants for practice. These word cards did not appear in the critical experiment. These word cards included experimental word pairs, semantically related filler word pairs, and semantically irrelevant filler word cards. During the practice, the experimenter did not give the participants any clues but asked them to judge which word pairs in the three cards were related and to report them orally. The instructions were: “You will be presented with three cards at the same time. You need to choose a card from these cards and judge which two words are related. Please report your judgement orally. I will give feedback whether it is correct or not according to your answer.” After the participants reported the results, the experimenter gave feedback on whether they were correct or not. When the experiment started, the participants received further instructions from the experimenter—“Please judge whether the words on the cards are relevant as soon as possible in the next experiment according to the feedback you received in the practice stage. If they are relevant, please mark them with a pen on the Braille engraved with ‘relevant’ on the answer sheet. Otherwise, please mark them on the Braille engraved with ‘irrelevant.’” Although there were two versions of the experimental material, each participant was randomly allocated only one version in the experiment.

### 2.3. Results and Analysis

Three participants with a correct rate less than or equal to 50% were excluded, and a total of 2.27% of the data were excluded. Because the 48 filler word pairs have no spatial relationship and they cannot answer our research questions, there was no additional analysis of non-experimental words (e.g., semantically related filler pairs and semantically unrelated filler pairs). Only the correct rate data of 16 experimental word pairs were included in the data analysis. The accuracy and standard deviation under each condition are shown in Table 1.

SPSS 26.0 was used to conduct a 2 × 2 × 2 analysis of variance on the correct rate data. The following criteria for determining the effect size come from Shen’s research [28]. The results showed that the main effect of the spatial relationship was significant *F*(1,125) = 405.754, *p* < 0.001, η^2^_p_ = 0.764 > 0.138 (large effect size), and the correct rate in the case of consistent positional relationship was significantly higher than that in the inconsistent case. The main effect of the grade was significant *F*(1,125) = 69.319, *p* < 0.001, η^2^_p_ = 0.357 > 0.138 (large effect size); the correct rate of participants who were junior school and above was higher than that of participants who were primary school. The main effect of participant type was not significant, *F*(1,125) = 1.614, *p* = 0.206, 0.01 < η^2^_p_ = 0.013 < 0.059 (small effect size), and there was no significant difference in the correct rate between congenitally blind participants and acquired blind participants. The interaction effect between spatial relationship and grade was not significant *F*(1,125) = 1.199, *p* = 0.276, η^2^_p_ = 0.009 < 0.01, and the interaction effect between spatial relationship and participant type was not significant *F*(1,125) = 0.046, *p* = 0.830, η^2^_p_ < 0.001; their three-factor interaction was also not *F*(1,125) = 0.217, *p* = 0.642, η^2^_p_ = 0.002 < 0.01. We also included age and gender as independent variables to analyze data. However, no interested results were significant.

The results of Experiment 1 using the semantic correlation judgment paradigm showed that blind students can automatically activate the spatial information of referents to represent knowledge through the tactile sensory channel when the visual sensory channel is blocked. The formation process of perceptual symbols is multi-channel. Moreover, the results also preliminarily showed that congenital blindness and acquired blindness did not affect the accuracy of knowledge representation of blind students. In contrast, blind students with more accumulated knowledge and experience have more advantages in knowledge representation. This suggested that learning may play an important role in the formation of perceptual symbols. Therefore, to further verify that visual experience and other channels play an equally important role in the knowledge representation of blind students, Experiment 2 refines the process of judging the positional relationship in Experiment 1 to study this proposition.

## 3. Experiment 2

### 3.1. Purpose

In Experiment 2, participants used tactile perception to select the candidate word and moved it to the test word card. This manipulation could examine whether the experience obtained and accumulated by the tactile sensory channel played an important role in the knowledge representation of blind children.

### 3.2. Methods

#### 3.2.1. Participants

The same batch of participants in Experiment 1 rested for ten minutes after completing the experiment and then proceeded to the test of Experiment 2.

#### 3.2.2. Experimental Design

A mixed experimental design of 4 (spatial relationship: upper-up, upper-down, lower-up, or lower-down) × 2 (grade: primary school vs. junior school and above) × 2 (participant type: congenital blind vs. acquired blindness) was adopted. The intra-group variable was the spatial relationship, the between-group variable was the grade and the participant type, and the dependent variable was the correct rate of the participant’s response.

#### 3.2.3. Experimental Materials

The selection criteria and process of all experimental materials were consistent with Experiment 1.

In Experiment 2, there was only one group of the material. To counterbalance potential influence from the differences of materials, four material subgroups were created for the experiment. Each material subgroup consisted of 4 sets of concrete words related to each other in position, which contained 4 words and 4 experimental word pairs. The 4 words were words with implied middle position (e.g., cup). Meanwhile, the 4 experimental word pairs were word pairs implying upper or lower spatial relationships (e.g., cup lid–cup mat). All material subgroups had the same 4 words and 4 experimental word pairs. The four subgroups together constituted one experimental group.

In the first subgroup, the words with implied middle positions were presented on the upper half of the test word card; the experimental word pairs were presented on the upper and lower half of the alternative word card. The correct word was on the upper half of the alternative word card, and the position type of this subgroup was recorded as “upper-up“. In the second subgroup, the words with implied middle positions were presented on the upper half of the test word card; the experimental word pairs were presented on the upper and lower half of the alternative word card. The correct words were on the lower half of the alternative word card; the position type of the subgroup was recorded as “upper-lower”. In the third subgroup, the words with the implied middle position were presented on the lower half of the test word card; the experimental word pairs were presented on the upper and lower half of the alternative word card. The correct word was on the upper half of the alternative word card; the position type of the subgroup was marked as “lower-up”. In the fourth group, the words with the implied middle position were presented on the lower half of the test word card, the experimental word pairs were presented on the upper and lower half of the alternative word card, and the correct word was on the lower half of the alternative word card, the position type of the subgroup was recorded as “lower-down”.

**Figure 3:**
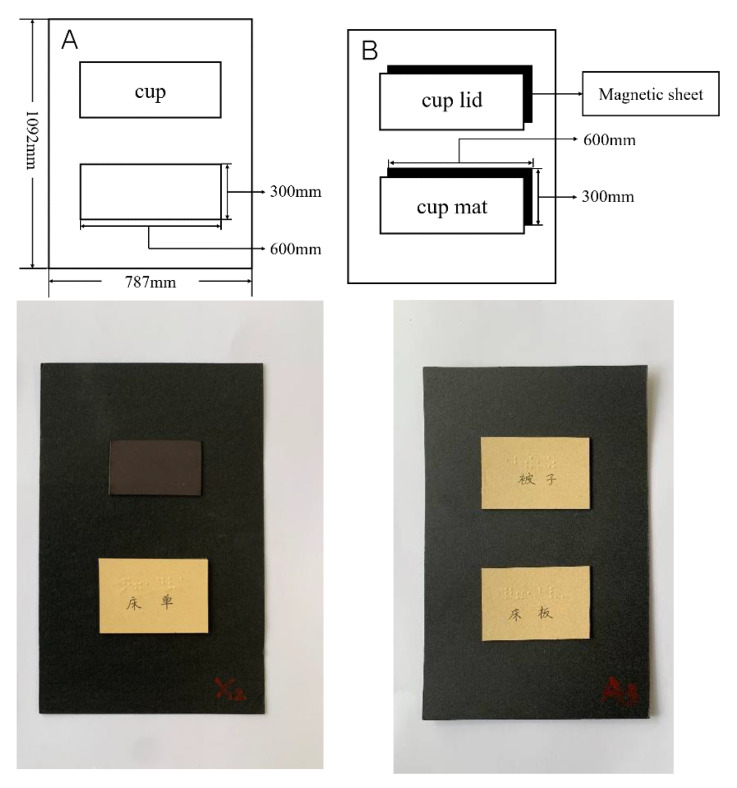
Schematic diagram of word cards in Experiment 2. (**A**) A test word card; (**B**) an alternative word card. In this example, the alternative words present “cup lid” and “cup mat” while the test word “cup” is presented on the upper of A. The correct answer is to select “cup mat” from B and place it in the blank space below the “cup” of A. Below the illustrations are the materials used in the experiment. In the two images: “床单” means “bed sheet” in Chinese, “被子” means “quilt”, “床板” means “bed board”.

The making process and specifications of all word cards were the same as Experiment 1. Words implying intermediate position information were pasted on the upper or lower half of the 787 × 1092 mm card stock. At the same time, the magnetic sheet was pasted on the remaining half of the card and a 600 × 300 mm blank card was pasted on the magnetic sheet. The experimental word pairs were cut into 600 × 300 mm cards and then the magnetic sheets were pasted on their back. The magnetic sheets were also pasted on the corresponding positions of the upper and lower half of another blank piece of cardboard. In the experiment, experimental word pairs can be adsorbed on the alternative word card and the test word card through the magnetic sheet. Please refer to Figure 3 for a schematic diagram of the word card.

#### 3.2.4. Experimental Procedure

In the experiment, participants needed to complete the group of experimental materials, of which four subgroups of matching word cards would be randomly presented to them. Participants needed to touch and read the words on the test word card and the alternative word card. Then, participants judged the implied spatial relationship and position of the words on the test word card as soon as possible. They needed to select a word from the alternative word card with the correct implied spatial relationship and paste it into the blank position of the test word card. For example, when a word with the implied positional relationship on the test word card was “cup” and it was presented in the upper part of the word card, participants needed to select a cup mat from the alternative word card and paste it into the blank part below the test word card. Before the critical experiment, participants needed to complete the practice part to be familiar with the experimental process. The test cards and alternative cards used in the practice session were different from the critical experiments. At the same time, participants would receive feedback on whether their responses were correct or not in the practice part. When participants reached a certain correct rate, the experiment started.

### 3.3. Results and Analysis

All participants had a correct rate of more than 50%, and no participants were excluded. Correct rate data for four different location type series were included in the data analysis. The accuracy and standard deviation under each condition are shown in Table 2.

The results of 4 × 2 × 2 analysis of variance on the correct rate data showed that the main effect of location type was significant, *F*(3,384) = 9.202, *p* < 0.001, 0.059 < η^2^_p_ = 0.067 < 0.138 (medium effect size). The accuracy of the four location types was analyzed by Bonferroni corrected post-hoc comparison. No significant differences were found between the upper-up location and the upper-down location (*p* > 0.05), and between the lower-down location and the lower-up location (*p* > 0.05). Meanwhile, significant differences appeared between the upper-up location and the lower-up location (*p* = 0.001), between the upper-up location and the lower-down location (*p* = 0.044), between the upper-down location and the lower-up location (*p* < 0.001), and between the upper-down location and the lower-down location (*p* = 0.004). Hence, the accuracy of the upper-up location and the upper-down location is higher than that of the lower-down location and the lower-up location.

The main effect of grade was significant *F*(3,384) = 50.349, *p* < 0.001, η^2^_p_ = 0.282 > 0.138 (large effect size). The correct rate of participants who were in junior high school and above was significantly higher than participants who were in primary school. The main effect of participant type was not significant *F*(3,384) = 0.798, *p* = 0.373, η^2^_p_ = 0.006 < 0.01, and there was no significant difference in the correct rate between congenitally blind participants and acquired blind participants (*p* > *0*.05). The interaction effect between spatial location and grade was not significant *F*(3,364) = 1.664, *p* = 0.178, 0.01 < η^2^_p_ = 0.013 < 0.059 (small effect size), and the interaction effect between spatial location and participant type was not significant *F*(3,364) = 0.201, *p* = 0.884, η^2^_p_ = 0.002 < 0.01; the three-factor interaction was also not significant *F*(3,364) = 0.922, *p* = 0.425, η^2^_p_ = 0.007 < 0.01. We analyzed age and gender as independent variables as well. However, no interested results were significant.

Experiment 2 showed that there was no significant difference in the way of knowledge representation between congenitally blind and acquired blind students. However, there were differences in the representation of knowledge in participants of different ages and the different spatial positions. This also suggested that the experience gained and accumulated through the tactile channel plays an important role in the knowledge representation of blind students.

## 4. Discussion

This study explored whether there is a perceptual symbol representation in the conceptual representation of congenitally blind and acquired blind individuals and the influence of perceptual experience acquired by tactile sensory channel on the conceptual representation of blind students. The significance of the main effect of the spatial relationship found in Experiment 1 proved that there is a perceptual symbol representation in the conceptual representation of the blind population. The significance of the main effect of the spatial relationship and grade in Experiment 2 further proved the important influence of perceptual experience on the conceptual representation of blind students. The present findings also have great implications. Specifically, blind children may use tactile perception to compensate for visual impairment. Therefore, language rehabilitation and language learning for blind children may recruit multiple perceptual channels to help them learn and represent language.

Previous studies have verified the perceptual symbol representation in the concrete concept representation. Moreover, the theory of perceptual symbols holds that perceptual symbols come from perceptual experience [14]. However, there were no clear theoretical views on how to construct perceptual symbol representation for individuals with perceptual channel defects and whether the participants with acquired blindness can still use the existing or establish new perceptual symbol representation after losing the visual sensory channel. Aiming at the question of whether blind students have perceptual symbol representation, this study used the paper-pencil test in Experiment 1 to examine the correct rate of the participants’ judgments on the semantic correlation of words presented in the upper and lower positions. The results showed that blind participants can still rely on the tactile sensory channel to complete the task of knowledge representation in the absence of visual channels, and there was no representation difference between congenital blindness and acquired blindness, but knowledge experience had a significant impact on the correct rate of task completion. In particular, under the condition of the same unguided practice stage, no matter the condition of spatial consistency or inconsistency, the individuals in the junior school stage always had a higher correct rate than the individuals in the primary school stage. The above results suggest that the perceptual symbol representation of blind students exists and may be similar to that of sighted people. In addition, perceptual experience may have a certain degree of influence on the conceptual representation of blind students. In Experiment 1, the fact that blind students can automatically activate the spatial information of referents for knowledge representation through the tactile sensory channel even when the visual sensory channel is blocked also shows the compensation phenomenon of other sensory channels. Experiment 1 was based on the word pair matching paradigm proposed by Zwaan and Yaxley [11] and used the spatial consistency effect to experimentally explore the perceptual symbol representation of concrete concepts of the blind. Compared with previous studies, this paper explored the perceptual symbol representation of blind people more directly and concretely and also discussed the compensation phenomenon of the perceptual channel in more detail from the perspective of the tactile sensory channel.

The tactile sensory channel is an important information transmission channel for the participants in the experiment. Information from tactile receptors is conducted by a system of lemniscus and anterolateral tracts to specific nucleuses of the thalamus, then to cortical sensory areas. Importantly, their distribution and range of cortical sensory representations are not immutable. Its size depends on internal developmental mechanisms and life experience, and is adjusted according to the importance of the function. Blind children usually use auditory and tactile sensory channels when they perceive information around them [16]. Thannhäuser also suggested that the tactile perception ability of blind children was superior to that of normal children. The differences in sensory cortex development between blind and sighted children may be related to cortical compensations after inactivation of the visual cortex [16].

From the above discussion on tactile sensory channel, we can see the importance of compensation of sensory and perceptual channels for blind students. Sensory channel compensation refers to a means to allow a person who has suffered sensory loss to make use of their remaining senses to perform functions normally carried out using the lost sense [29]. Braille is the vehicle for blind people to read and interact with their fingers instead of vision. Due to the absence of visual sensory channel, the input from vision will be compensated by inputs from other channels. This process changes the plasticity of the brain, i.e., the neuroplasticity [16]. People receive perceptual information from multiple sensory channels. For example, people use both touch and vision to perceive the shape of an object. When one wants to identify the specific location of a sound source, s/he uses vision and auditory sense to identify the location. Therefore, previous studies pointed out that the involvement of such redundant sensory perception channels enables the brain to compensate for the absence of some sensory channels [30]. Pasqualotto et al. showed that people who were born blind or lost sight at an early age tended to perform better in finer tone discrimination and sound localization, tactile discrimination, speech discrimination, and verbal recall [31,32]. Changes in neuroplasticity also affected areas of the brain once specialized in visual tasks. Previous neuroimaging studies found that the visual occipital cortex is responsible for processing tactile and auditory information, as well as higher cognitive functions such as grammar and language processing in blind people [33]. Blind adults’ speech comprehension could activate the striate and extra-striate regions of the visual cortex [33]. Meanwhile, speech comprehension in blind people also activated classical perisylvian language areas in the left hemisphere in speech processing [34] and auditory verb generation [35,36], which were the same as typical adults. The exploration of compensatory phenomena of sensory channels will also contribute to the formulation of more appropriate language learning and rehabilitation programs for blind children. Parents and educators of blind children can consider fully mobilizing other sensory channels, enriching the language learning methods and contents of blind children from the perspective of sound, touch and even taste. These can increase the input channels of language information so that blind students can obtain as much language information as possible, thus improving the efficiency and results of learning.

In Experiment 2 of this study, a word-card-pairing experiment was set up to explore whether the experience gained from the tactile sensory channel played a key role in the knowledge representation of blind students. The participants were required to reasonably select and place the alternative words according to the word meaning and presentation position of the test words. The results showed that the main effect of the spatial relationship was still significant. There was also no difference in representation between congenital blindness and acquired blindness. However, knowledge representation was different in different grades. This suggests that knowledge and experience may have a key influence on the knowledge representation of blind students.

Therefore, how do knowledge and experience affect the knowledge representation of blind students? Barbara Treccani and Claudio Mulatti suggested that the effect of conceptual compatibility on the consistency of spatial position relation of words may not be due to the simulation of perceptual and motor processes, but to task-related factors [37]. In simple terms, the task is to give the “labels” to stimulus and responses. Meanwhile, the compatibility effect of the spatial concept is caused by the overlapping of stimulus and response “labels”. In the case of overlap, participants often receive answers more accurately and faster. In this study, participants did not receive direct experimental guidance before conducting Experiments 1 and 2. They could only rely on the feedback from the experimenters on their behavior during the practice phase to obtain corresponding knowledge and experience to make judgments. According to the content of the viewpoints driven by the above task-related factors, the process of knowledge and experience formation in the practice phase can be equivalent to the process of labeling stimuli and responses. Hence, in the formal experiment, when the participants used the knowledge and experience formed in the practice stage to complete the task, there would be different understandings and choices of the participants facing different spatial position relations. In Experiment 2, the difference in the accuracy of different position types was influenced by the knowledge and experience of the participants.

Why was there no difference in representation between congenital blindness and acquired blindness in both Experiments 1 and 2? In fact, this result was actually not what we expected at the beginning. The critical period of tactile sense most likely occurs earlier than the critical period of vision or hearing. Reviewing several studies of tactile stimulation in animals, Casler noted that the neuropathic skin may have been one of the first cells to fully develop and activate the formation of reticular structures [38]. Later, these channels will have to compete with others and become less effective. It has also been found that participants with early blindness (before 6 months) have better immediate memory of the place where they were stimulated by touch than participants with advanced blindness (3 years later) or normal vision [39]. This suggested that some of the tactile experiences that were characteristic of the early blind participants were crucial to their later performance. In another defect test of sensory deprivation in chimpanzees, the importance of early touch and associated motor information for full development was also indicated [40]. All of the above studies indicated that congenitally blind participants have an overall advantage over acquired blind participants in obtaining information through tactile sensory channel. However, this was not the case in the two experiments in this study.

Previous studies hold that differences in spatial location perception used by blind people are mainly due to the different weights of internal and external coordinate systems [41,42,43,44]. The internal coordinate system (also known as the anatomical reference frame) takes the human body as the reference standard for the spatial location of other objects (e.g., in the palm, on the back of the hand). In contrast, the external coordinate system is not egocentric. Its spatial frame is composed of external objects (e.g., above the table, below the chair) [45]. Due to a lack of vision, blind people preferentially rely on the internal coordinate system when processing tactile spatial information [41,42,43,44,46]. The internal coordinate system locates stimuli by recognizing and sensing the position of objects using the tactile sensation [47]. The internal coordinate system can also specify the location of target objects in the processing of memorized information [43]. Crollen et al. proved that the internal coordinate system is very important for blind people by comparing the different performances of sighted participants and congenital blind participants in the tactile temporal order judgment (TOJ) task and auditory Simon task and its existence enables the blind to better think about spatial issues [19]. It can be said that there are perceptual differences among blind groups, but the differences are not affected by congenital or acquired blindness. The proportion between internal and external coordinates in blind individuals and the call to the internal and external coordinate systems when facing specific task instructions may be the key to affecting the symbolic representation of blind people’s perception.

Experiment 2 found that location type affected participants’ responses. These results might be interpreted by the Sapir–Whorf hypothesis. This hypothesis holds that language may shape people’s thinking [48]. In a long history, traditional Chinese was written in the vertical direction from the upper to the lower location [49]. In this way, Chinese may also affect the spatial representation of words in the vertical axis. Previous studies also found that Mandarin speakers usually described time using the vertical axis while English speakers usually described time using the horizontal axis [50]. This result suggested that Mandarin speakers may tend to represent concepts in the vertical axis. Therefore, in the present study, when participants read words in the vertical direction on the test word card, they responded better to the upper words than the lower words.

At the same time, Wang Ruiming’s reproduction and improvement of the Zwaan word-pair-matching paradigm in Chinese users showed that participants would automatically activate the spatial information of referents in the semantic correlation judgment task [12]. Based on the above experiments, this experiment further expanded and showed that blind Chinese students have perceptual symbol representation despite the lack of visual sensory channel. Knowledge and experience played an important role in it.

Humans’ various senses are the gate of human knowledge. They play a very important role in feeling, perceiving, and understanding things. Various studies have pointed out many psychosocial problems and low levels of adaptive ability in blind people [51]. People’s emotional development and social skills are learned through their relationships with others, among which vision and hearing play an important role in many skills, while blind people have greater difficulty in acquiring such experiences due to defects in visual sensory channel. Tröster and Brambring in a previous study on the social and emotional development of blind children found that blind or semi-blind children had many limitations in facial expressions [52]. Due to visual impairment, they have very limited opportunities to contact facial expressions, which makes them have many difficulties in daily social communication. It also easily causes social pressure, thus endangering their mental health. 

## 5. Limitations and Future Research

There were still shortcomings in this study. Due to the special nature of the participants, when the responses of the participants could not be collected, the current experimental data was mainly based on the correct rate. The absence of response-time data was not conducive to revealing the dynamic process of cognitive representation in blind people. The particularity of the participants themselves had many effects on the acquisition of their reaction time. The acquisition of reaction time of blind people was different from that of sighted participants. Because they were blind, their reaction was slower than sighted children. They needed to rely on the tactile touch of the hand to feel the experimental materials and touch the position where the stimulus should be. Therefore, there were many cognitive processing processes irrelevant to our research. The data of reaction time included too many cognitive processes, which made them unsuitable for further analysis. Compared with the reaction time, the accuracy of the participants in this experiment was more in line with the exploration of the main problems of the article. Although the lack of reaction time data was not conducive to our analysis of the dynamic process of cognitive representation, the accuracy data was also in line with the analysis theme of this article.

This study mainly discussed the representation of concrete concepts by blind students. Therefore, future research can further explore the representation of abstract concepts by blind students. In addition, the current research only measures the representation of words that blind students have acquired, so how will the blind students represent newly acquired words? As mentioned above, the difference in representation between blind people comes from the weight difference between internal and external coordination systems. Therefore, what are the factors that regulate this weight difference?

## 6. Conclusions

This study mainly explored the existence of perceptual symbol representations in the blind population and the influence of experiences acquired by the tactile sensory channel on the knowledge representation of blind people. The results found that blind students can still express knowledge by activating the spatial information of referents through the tactile sensory channel when the visual sensory channel is blocked, and the knowledge experience obtained by other channels (mainly the tactile sensory channel) is in the knowledge of the blind students. The experimental results supported the theory of perceptual symbols and reflected the flexibility and diversity of perceptual symbol representation. Moreover, the above findings not only provided new evidence and research directions for the theory of perceptual symbols but also provide a basic reference for the field of language rehabilitation and learning for blind students. Our research is expected to further understand and improve the situation of blind children by studying their perceptual symbol representation, providing feasible training programs for their families and educators, reducing their anxiety in social interaction, and thus preventing possible mental health problems in the future.

## Figures and Tables

**Table 1:** Correct rate, significance and effect size of participants’ responses under different grades, participant types, and spatial location (M ± SD).

Grade	Participant Type	Consistent Spatial Relationship	Inconsistent Spatial Relationship	*p*	η^2^_p_
Primary school	Congenital blindness	0.85 ± 0.09	0.68 ± 0.07	<0.001	0.485
	Acquired blindness	0.87 ± 0.09	0.71 ± 0.11	<0.001	0.410
Junior school and above	Congenital blindness	0.95 ± 0.06	0.81 ± 0.09	<0.001	0.505
	Acquired blindness	0.96 ± 0.06	0.81 ± 0.10	<0.001	0.417

**Table 2:** Correct rate, significance and effect size of participants’ responses under different grades, participant types, and spatial location (M ± SD).

Grade	Participant Type	Upper-Up	Upper-Lower	Lower-Up	Lower-Down	*p*	η^2^_p_
Primary school	Congenital blindness	0.83 ± 0.15	0.82 ± 0.17	0.76 ± 0.23	0.76 ± 0.21	0.008	0.089
	Acquired blindness	0.84 ± 0.14	0.86 ± 0.15	0.74 ± 0.19	0.76 ± 0.14	0.005	0.095
Junior school and above	Congenital blindness	0.93 ± 0.11	0.95 ± 0.10	0.85 ± 0.16	0.91 ± 0.15	0.029	0.069
	Acquired blindness	0.92 ± 0.12	0.94 ± 0.11	0.91 ± 0.12	0.90 ± 0.12	0.838	0.007

## Data Availability

The data that support the findings of this study are available from the corresponding author upon reasonable request.
